# Prescribing patterns of fall risk-increasing drugs in older adults hospitalized for heart failure

**DOI:** 10.1186/s12872-023-03401-w

**Published:** 2023-07-26

**Authors:** Esther Liu, Musarrat Nahid, Mahad Musse, Ligong Chen, Sarah N. Hilmer, Andrew Zullo, Min Ji Kwak, Mark Lachs, Emily B. Levitan, Monika M. Safford, Parag Goyal

**Affiliations:** 1 Department of Medicine, Weill Cornell Medicine, Weill Medical College of Cornell University, 525 East 68Th Street, New York, NY 10021 USA; 2 University of Alabama at Birmingham, Birmingham, AL USA; 3 The University of Sydney and Royal North Shore Hospital, Sydney, NSW Australia; 4 Brown University School of Public Health, Providence, USA; 5 The University of Texas, Houston, TX USA

**Keywords:** Falls, Heart failure, Fall risk-increasing drugs

## Abstract

**Background:**

Older adults hospitalized for heart failure (HF) are at risk for falls after discharge. One modifiable contributor to falls is fall risk-increasing drugs (FRIDs). However, the prevalence of FRIDs among older adults hospitalized for HF is unknown. We describe patterns of FRIDs use and examine predictors of a high FRID burden.

**Methods:**

We used the national biracial REasons for Geographic and Racial Differences in Stroke (REGARDS) study, a prospective cohort recruited from 2003–2007. We included REGARDS participants aged ≥ 65 years discharged alive after a HF hospitalization from 2003–2017. We determined FRIDs –cardiovascular (CV) and non-cardiovascular (non-CV) medications – at admission and discharge from chart abstraction of HF hospitalizations. We examined the predictors of a high FRID burden at discharge via modified Poisson regression with robust standard errors.

**Results:**

Among 1147 participants (46.5% women, mean age 77.6 years) hospitalized at 676 hospitals, 94% were taking at least 1 FRID at admission and 99% were prescribed at least 1 FRID at discharge. The prevalence of CV FRIDs was 92% at admission and 98% at discharge, and the prevalence of non-CV FRIDs was 32% at admission and discharge. The most common CV FRID at admission (88%) and discharge (93%) were antihypertensives; the most common agents were beta blockers (61% at admission, 75% at discharge), angiotensin-converting enzyme inhibitors (36% vs. 42%), and calcium channel blockers (32% vs. 28%). Loop diuretics had the greatest change in prevalence (53% vs. 72%). More than half of the cohort (54%) had a high FRID burden (Agency for Healthcare Research and Quality (AHRQ) score ≥ 6), indicating high falls risk after discharge. In a multivariable Poisson regression analysis, the factors strongly associated with a high FRID burden at discharge included hypertension (PR: 1.41, 95% CI: 1.20, 1.65), mood disorder (PR: 1.24, 95% CI: 1.10, 1.38), and hyperpolypharmacy (PR: 1.88, 95% CI: 1.64, 2.14).

**Conclusions:**

FRID use was nearly universal among older adults hospitalized for HF; more than half had a high FRID burden at discharge. Further work is needed to guide the management of a common clinical conundrum whereby guideline indications for treating HF may contribute to an increased risk for falls.

**Supplementary Information:**

The online version contains supplementary material available at 10.1186/s12872-023-03401-w.

## Introduction

Falls are a concern among older adults given their impacton morbidity and mortality [1]. Almost one-third of older adults who fall suffer physical harm [2, 3]. Resulting hip fractures are especially relevant as they lead to decreased life expectancy, diminished functional capacity, and loss of independence [4, 5]. Older adults with HF are predisposed to a high risk for falls for multiple reasons. These include age-related physiologic alterations, such as decreased autonomic reflexes, reduced adrenergic responsiveness, and intravascular volume contraction, which contribute to orthostatic hypotension [6]; arrhythmias which can lead to impaired cardiac output and cardiac syncope [7]; and geriatric conditions such as cognitive impairment and frailty, predispose older adults to falls [8, 9]. Moreover, being in the hospital is a major risk factor for falls [10], and can be exacerbated by delirium [11]. Finally, polypharmacy, which is nearly universal in older adults hospitalized for HF, is also a major risk factor for falls [12–14]. Despite these risk factors, older adults are often prescribed HF-specific medications in addition to medications for other conditions which increase the risk for hypotension, dizziness, and subsequent falls [12, 15, 16].

Consequently, clinicians often face a conundrum when caring for older adults hospitalized for HF—medications to treat HF and other conditions can improve long-term outcomes, but might do so at an increased risk of falls. An improved understanding of the potentially modifiable contributors to fall risk in older adults hospitalized for HF could inform future intervention development to address this conundrum. Indeed, a modifiable contributor to falls could be the use of fall risk-increasing drugs (FRIDs), a group of medications with the potential to increase the risk of falls through effects on the cardiovascular (CV) and central nervous systems [17–19]. To our knowledge, the prevalence of FRIDs among older adults hospitalized for HF has not been described. To address this knowledge gap and create a foundation for future work to reduce risk of falls in this population, we characterized the prescribing patterns of FRIDs at the time of admission and discharge for a HF hospitalization, and examined the determinants of FRIDs prescribing at hospital discharge.

## Methods

### Study population

The REasons for Geographic and Racial Differences in Stroke (REGARDS) cohort study is a prospective cohort study that includes 30,239 White and Black community-dwelling men and women aged ≥ 45 years at enrollment recruited between 2003–2007 from all 48 contiguous states of the United States with ongoing follow-up [20]. The REGARDS study was originally designed to determine antecedents of racial and geographic differences in stroke mortality in the United States, and has since been used to study CV diseases including HF [12, 15, 21, 22]. Participants were randomly sampled, with oversampling in the Southeastern US and of Black participants. Participant information such as demographics and health behaviors were elicited at baseline through computer assisted telephone interviews and an in-home visit. CV outcomes and related hospitalizations are detected every 6 months via telephone and retrieval of medical records. Two expert clinician adjudicators determine whether hospitalizations are caused by HF based on review of medical records from the hospitalization; a third adjudicator is employed to resolve any disagreements. The institutional review boards of all collaborating institutions approved the REGARDS study protocol and all participants provided written informed consent at the time of enrollment.

For this study, we studied participants aged ≥ 65 years who were discharged alive after experiencing an adjudicated HF hospitalization between 2003 and 2017. We excluded participants referred to hospice at hospital discharge and excluded participants who did not have medication data at both hospital admission and discharge. In the case of multiple hospitalizations for a participant, we examined the first hospitalization.

### Data sources

We utilized data from three merged datasets: 1) the REGARDS study baseline assessment and periodic cognitive assessments; 2) medical record chart abstraction from adjudicated HF hospitalizations within REGARDS; 3) the American Hospital Association (AHA) Annual Survey Database, which contains information on > 6,500 hospitals across the US.

Baseline characteristics included age, sex, self-identified race, income, education, functional impairment (Physical Component Summary score < 40 from the Short Form 12-item questionnaire [23]. Patients were also asked about a history of falls, defined as at least one self-reported fall in the prior year. The 6-item screener, a short assessment of cognitive functioning, was administered during follow-up calls every 2 years; the cognitive assessment completed most closely and prior to the hospitalization was used, with cognitive impairment defined as a 6-item screener score < 5 [24].

Chart abstraction included data from admission notes, progress notes, discharge summaries, and medication reconciliation reports. We abstracted patients’ medical conditions, admission and discharge vital signs, laboratory values, echocardiogram parameters such as left ventricular ejection fraction (LVEF), medications prescribed on admission and discharge, and hospitalization factors such as length of stay, intensive care unit (ICU) stay, and discharge disposition. HF with preserved ejection fraction (HFpEF) was defined as LVEF ≥ 50% or a qualitative description of normal systolic function [25], and HF with a reduced ejection fraction (HFrEF) was defined as LVEF < 50% or a qualitative description of abnormal systolic function [26]. We grouped patients with HF with a mildly reduced ejection fraction (HFmrEF) with those with HFrEF given shared pathophysiology and response to treatment [26].

AHA Annual Survey data from 2003–2017 included hospital size, where a small hospital size was defined as < 200 beds; and academic/teaching status, defined as inclusion in the Association of American Medical Colleges Council of Teaching Hospitals and Health Systems or certification by the Accreditation Council for Graduate Medical Education.

### Fall risk-increasing medications (FRIDs)

We identified fall risk-increasing medications (FRIDs) as medications associated with increased fall risk based on comprehensive systematic reviews and meta-analyses [18, 19, 27–29]. We classified FRIDs into two main categorizations: CV FRIDs and non-CV FRIDs. CV FRIDs included antihypertensives (some of which are classically described as guideline-directed medical therapy for HF), including beta-blockers, renin-angiotensin system inhibitors, calcium channel blockers, nitrates, alpha blockers, vasodilators, alpha agonists, digoxin, and diuretics (thiazides, loop diuretics, potassium-sparing diuretics). Non-CV FRIDs included antidepressants, benzodiazepines, opioids, antiepileptics, and antipsychotics. Classification of CV vs. non-CV was based on a classification scheme previously used and primarily based on the Multum Lexicon Drug Database [12, 30].

To quantify FRID burden at hospital discharge, we used the Agency for Healthcare Research and Quality (AHRQ) medication-based fall risk tool [31]. This tool provides a weighted risk score for falls based on the medication regimen. To calculate the AHRQ score, each medication is assigned a score (higher scores indicate higher risk for falls), and then scores are then summed to generate a weighted score. Medication scores were calculated as follows: antihypertensives = 2, digoxin = 2, loop diuretics = 1. For non-CV FRIDs, drugs were assigned a score of 3, with the exception of antidepressants which were assigned a score of 2.

### Statistical analysis

To summarize participant and hospital characteristics, we calculated medians and interquartile ranges (IQR) for continuous variables and percentages for categorical variables. We also calculated the prevalence and median counts for all FRIDs, CV FRIDs, and non-CV FRIDs at admission and at discharge; and calculated the change between admission and discharge.

We calculated the prevalence of participants with a high FRID burden, and specified a multivariable modified Poisson regression model with robust standard errors to estimate prevalence ratios with 95% confidence intervals (CIs) to identify predictors of a high FRID burden at hospital discharge. High FRID burden was defined by an AHRQ medication-based fall risk score of ≥ 6, which is a threshold indicating excess risk [31, 32]. Candidate covariates in the model included: socio-demographics (age, sex, race, income, education), HF subtype (HFrEF vs. HFpEF), comorbid conditions for which FRIDs may be prescribed (hypertension, atrial fibrillation/atrial flutter, mood disorder, osteoarthritis, rheumatoid arthritis, and cancer), discharge medications that may impact FRIDs prescribing (anticoagulants), geriatric conditions (functional impairment, cognitive impairment, history of falls in the year prior to REGARDS study enrollment, hyperpolypharmacy – defined as the use of at least 10 medications at discharge), hospitalization factors (length of stay, intensive care unit (ICU) stay, geriatric/palliative involvement, discharge disposition), and hospital characteristics (teaching status, hospital size). A p-value < 0.05 was considered significant. To account for missing covariate values, we used multiple imputation via chained equations [33]. Of note, the covariates with the highest degree of missingness were HF subtype (35%), cognitive impairment (27%), and income (13%). We managed the data in SAS version 9.4 (SAS Institute, Cary, NC) and performed statistical analysis using STATA version 17 (StataCorp LLC, College Station, TX).

## Results

We examined 1147 unique participants hospitalized for HF. The median age was 78 years (IQR: 72,83 years), 47% were women, and 40% were Black persons (Table 1). The most common comorbid condition was hypertension (79%). The median number of medications taken at hospital admission was 9 (IQR: 6, 12) and at discharge was 10 (IQR: 8, 13). The prevalence of hyperpolypharmacy was 45% at admission and 57% at discharge.

**Table 1:** Baseline characteristics according to FRIDs use at admission

Characteristics	All	FRIDS	
		Absent	Present
N	1147	67	1080
Age, years	77.6 (72.2, 83.1)	76.8 (73.8, 82.4)	77.73 (72.14, 83.23)
Black	456 (39.8%)	18 (26.9%)	438 (40.6%)
Female	533 (46.5%)	26 (38.8%)	507 (46.9%)
Education less than high school	243 (21.2%)	16 (23.9%)	227 (21.0%)
Income < $20,000	286 (28.5%)	15 (25.0%)	271 (28.7%)
**Clinical Characteristics**			
HFrEF	395 (52.8%)	20 (48.8%)	375 (53.0%)
Hypertension	899 (78.5%)	26 (39.4%)	873 (80.9%)
Atrial fibrillation/atrial flutter	482 (42.0%)	29 (43.3%)	453 (41.9%)
Mood disorder	176 (15.3%)	3 (4.5%)	173 (16.0%)
Osteoarthritis	298 (26.0%)	11 (16.4%)	287 (26.6%)
Rheumatoid arthritis	27 (2.4%)	5 (7.5%)	22 (2.0%)
Cancer	219 (19.1%)	9 (13.4%)	210 (19.4%)
Anticoagulant use	352 (30.7%)	24 (35.8%)	328 (30.4%)
Hyperpolypharmacy (at discharge)	657 (57.3%)	20 (29.9%)	637 (59.0%)
Functional impairment	461 (43.0%)	13 (21.0%)	448 (44.3%)
Cognitive impairment	125 (14.9%)	6 (12.8%)	119 (15.0%)
History of falls (in the past year)	233 (20.4%)	9 (13.4%)	224 (20.8%)
**Hospitalization Factors**			
Length of stay (days)	5.0 (3.0, 8.0)	5.5 (4.0, 9.0)	5.00 (3.00, 8.00)
ICU stay during hospitalization	211 (18.4%)	20 (30.3%)	191 (17.7%)
Geriatric/palliative involvement	13 (1.1%)	1 (1.5%)	12 (1.1%)
Disposition			
Home	908 (79.2%)	52 (77.6%)	856 (79.3%)
Institution	185 (16.1%)	9 (13.4%)	176 (16.3%)
Alive, unknown disposition	54 (4.7%)	6 (9.0%)	48 (4.4%)
**Hospital Characteristics**			
Teaching status	527 (51.5%)	31 (50.0%)	496 (51.6%)
Small hospital size (< 200 beds)	262 (25.1%)	13 (21.0%)	249 (25.4%)

Values are n (%), unless otherwise indicated

*Abbreviations*: *HF* Heart failure, *HFrEF* Heart failure with reduced ejection fraction, *ICU* Intensive care unit

**Fig. 1:**
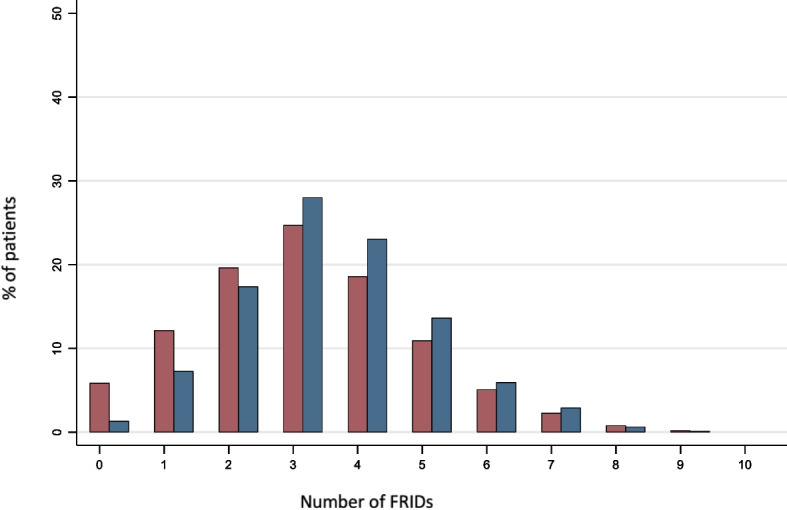
Frequency of FRID Counts at Admission (red) and Discharge (blue). FRIDs = fall risk-increasing drugs

The prevalence of FRIDs use at hospital admission was 94% and at discharge was 99% (Tables 1 and 2, Supplemental Tables 1-2). The median number of FRIDs at both hospital admission and discharge was 3 (IQR: 2, 4). At admission, 37% took at least 4 FRIDS; and at discharge, 47% took at least 4 FRIDs (Fig. 1). Nearly half (42%) of patients were taking at least one additional FRID at discharge compared to admission. The most commonly initiated FRIDs (present on discharge, absent on admission) were loop diuretics (26%) and beta blockers (18%). The two most commonly discontinued FRIDs (present on admission, absent on discharge) were thiazides (10%) and calcium channel blockers (CCB) (10%). These patterns were similar among those with HFrEF and HFpEF (Supplemental Fig. 1-2 and Supplemental Table 3).

**Table 2:** Baseline characteristics according to FRIDs use at discharge

Characteristics	FRIDs	
	Absent	Present
N	15	1132
Age, years	76.6 (70.3, 83.0)	77.7 (72.3, 83.2)
Black	5 (33.3%)	451 (39.8%)
Female	9 (60.0%)	524 (46.3%)
Education less than high school	3 (20.0%)	240 (21.2%)
Income < $20,000	5 (41.7%)	281 (28.4%)
**Clinical Characteristics**		
HFrEF	2 (16.7%)	393 (53.4%)
Hypertension	6 (40.0%)	893 (79.0%)
Atrial fibrillation/atrial flutter	7 (46.7%)	475 (42.0%)
Mood disorder	4 (26.7%)	172 (15.2%)
Osteoarthritis	4 (26.7%)	294 (26.0%)
Rheumatoid arthritis	1 (6.7%)	26 (2.3%)
Cancer	3 (20.0%)	216 (19.1%)
Anticoagulant use	5 (33.3%)	347 (30.7%)
Hyperpolypharmacy (at discharge)	3 (20.0%)	654 (57.8%)
Functional impairment	6 (42.9%)	455 (43.0%)
Cognitive impairment	2 (18.2%)	123 (14.8%)
History of falls (in the past year)	2 (13.3%)	231 (20.5%)
**Hospitalization Factors**		
Length of stay (days)	7.0 (4.0, 14.0)	5.00 (3.00, 8.00)
ICU stay during hospitalization	8 (53.3%)	203 (18.0%)
Geriatric/palliative involvement	1 (6.7%)	12 (1.1%)
Disposition		
Home	9 (60.0%)	899 (79.4%)
Institution	4 (26.7%)	181 (16.0%)
Alive, unknown disposition	2 (13.3%)	52 (4.6%)
**Hospital Characteristics**		
Teaching status	7 (50.0%)	520 (51.5%)
Small hospital size (< 200 beds)	3 (21.4%)	259 (25.2%)

Values are n (%), unless otherwise indicated

*Abbreviations*: *HF* heart failure, *HFrEF* Heart failure with reduced ejection fraction, *ICU* Intensive care unit

**Fig. 2:**
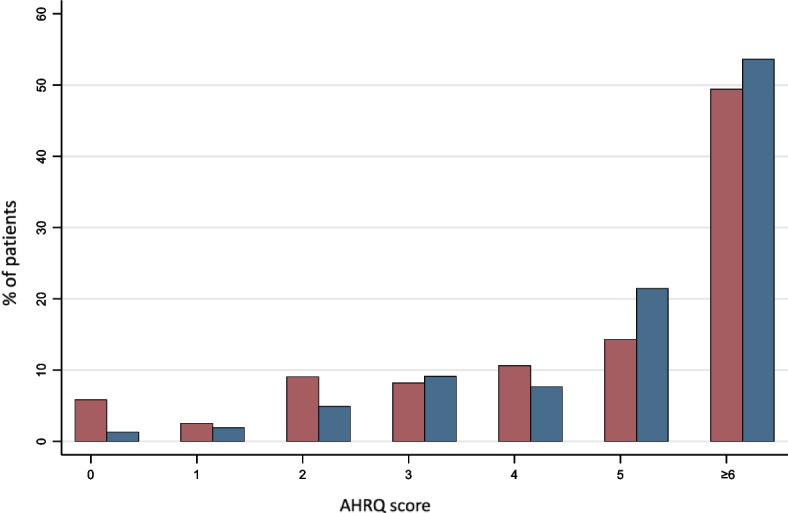
Frequency of AHRQ Fall Risk Score at Admission (red) and Discharge (blue). AHRQ = Agency for Healthcare Research and Quality

The majority of FRIDs were CV in nature—on average, 82% of FRIDs were CV in nature at admission; and 88% of FRIDs were CV in nature at discharge. The proportion of individuals taking CV FRIDs increased from 92% at admission to 98% at hospital discharge (Table 3). The median count of CV FRIDs was 3 at admission and discharge (IQR: 2, 4). Between admission and discharge, 41% had at least one CV FRID added to their medication regimen. Antihypertensives were the most common class of CV FRID at both admission (88%) and discharge (93%). Common CV FRIDs included beta blockers (61% at admission, 75% at discharge), ACEi (36% at admission, 42% at discharge), and CCB (32% at admission, and 28% at discharge). The CV FRID with the greatest change in prevalence between admission and discharge was loop diuretics, which increased from 53% at admission to 72% at discharge (Table 3). These patterns were similar among those with HFrEF and HFpEF (Supplemental Table 4–5).

**Table 3:** Prevalence of FRIDs use at hospital admission and discharge

	**Admission**	**Discharge**	**Change**
**All FRIDs**	1080 (94)	1132 (99)	5%
**CV FRIDs**	1060 (92)	1122 (98)	6%
**Antihypertensives**	1005 (88)	1072 (93)	5%
Beta blockers	700 (61)	863 (75)	14%
ACE inhibitors	411 (36)	480 (42)	6%
CCBs	369 (32)	325 (28)	-4%
Nitrates	179 (16)	250 (22)	6%
Alpha blockers	230 (20)	215 (19)	1%
ARBs	228 (20)	203 (18)	-2%
Vasodilators	99 (9)	152 (13)	4%
Aldosterone antagonists	81 (7)	148 (13)	6%
Thiazides	183 (16)	90 (8)	-8%
Potassium-sparing diuretics	23 (2)	8 (1)	-1%
Alpha agonists	7 (1)	9 (1)	-
Loop diuretics	612 (53)	829 (72)	19%
Digoxin	141 (12)	185 (16)	4%
**Non-CV FRIDs**	369 (32)	368 (32)	-
**Antidepressants**	234 (20)	243 (21)	1%
SSRI	157 (14)	165 (14)	-
TCA	46 (4)	46 (4)	-
SNRI	28 (2)	30 (3)	1%
Trazodone	10 (1)	12 (1)	-
NDRI	8 (1)	8 (1)	-
Benzodiazepines	93 (8)	87 (8)	-
Opioids	97 (8)	71 (6)	-2%
Antiepileptics	30 (3)	34 (3)	-
**Antipsychotics**	19 (2)	29 (2)	-
Atypical	16 (1)	24 (2)	1%
Typical	3 (0)	5 (0)	-

*Abbreviations*: *FRIDs* Fall risk-increasing drugs, *CV* Cardiovascular, *ACEi* Angiotensin-converting enzyme inhibitors, *CCB* Calcium channel blocker, *ARB* Angiotensin receptor blocker, *Non-CV* Non-cardiovascular, *SSRI* Selective serotonin reuptake inhibitor, *TCA* Tricyclic antidepressant, *SNRI* Serotonin and norepinephrine reuptake inhibitor, *NDRI* Norepinephrine and dopamine reuptake inhibitor

The prevalence of non-CV FRIDs was 32% at both admission and discharge. At admission, 23% took 1 non-CV FRID, 7% took 2 non-CV FRIDs, and 2% took 3 non-CV FRIDs. At discharge, 24% took 1 non-CV FRID, 7% took 2 non-CV FRIDs, and 2% took 3 non-CV FRIDs. Between admission and discharge, 8% had at least one non-CV FRID initiated. The most common class of non-CV FRID at both admission (20%) and discharge (21%) was antidepressants. The non-CV FRID with the greatest change between admission and discharge was opioids, which decreased from 8 to 6% (Table 3). These patterns were similar among those with HFrEF and HFpEF (Supplemental Tables 4–5).

At discharge, 54% of the cohort had an AHRQ score ≥ 6, indicating a high FRID burden (Fig. 2). The prevalence was comparable for HFpEF and HFrEF (Supplemental Fig. 3–4). In a multivariable analysis, factors most strongly associated with a high FRID burden at discharge were hypertension (PR: 1.41, 95% CI: 1.20, 1.65), mood disorder (PR: 1.24, 95% CI: 1.10, 1.38), and hyperpolypharmacy (PR: 1.88, 95% CI: 1.64, 2.14) (Table 4).

**Table 4:** Predictors of high FRID burden at discharge

	**PR**	**95% CI**	***P*** **value**
Age (per 10 years)	0.92	(0.86, 0.99)	0.04
Black	1.11	(0.99, 1.24)	0.09
Female	1.02	(0.91, 1.14)	0.74
Education less than high school	0.98	(0.86, 1.11)	0.76
Income less than $20,000	1.04	(0.91, 1.19)	0.57
HFrEF	1.03	(0.91, 1.16)	0.66
Hypertension	1.41	(1.20, 1.65)	< 0.001
Atrial fibrillation/atrial flutter	1.00	(0.88, 1.14)	0.98
Mood disorder	1.24	(1.10, 1.38)	< 0.001
Osteoarthritis	1.08	(0.97, 1.21)	0.18
Rheumatoid arthritis	0.73	(0.47, 1.13)	0.15
Cancer	1.07	(0.95, 1.21)	0.27
Anticoagulation use	0.94	(0.83, 1.08)	0.41
Functional impairment	1.09	(0.98, 1.21)	0.12
Cognitive impairment	1.13	(0.96, 1.33)	0.15
History of falls	1.04	(0.92, 1.18)	0.49
Hyperpolypharmacy (at discharge)	1.88	(1.64, 2.14)	< 0.001
Length of stay (per day)	0.99	(0.99, 1.01)	0.50
ICU stay during hospitalization	0.97	(0.84, 1.12)	0.71
Geriatric/palliative involvement	0.85	(0.43, 1.69)	0.65
Discharge to Facility	0.89	(0.76, 1.03)	0.13
Discharged Alive but unknown disposition	1.20	(0.97, 1.45)	0.10
Teaching hospital	0.94	(0.83, 1.06)	0.32
Small hospital (< 200 beds)	1.05	(0.92, 1.20)	0.47

*Abbreviations*: *PR* Prevalence ratio, *CI* Confidence interval, *HFrEF* Heart failure with reduced ejection fraction, *ICU* Intensive care unit

## Discussion

Older adults with HF are at a high risk of falls, which can lead to substantial physical, psychological, and social consequences. This supports the importance of understanding prescribing patterns of agents that can contribute to this risk (FRIDs). This study is the first to our knowledge to examine prescribing patterns of FRIDs in this population. From this study, we found that FRIDs are nearly universal among older adults hospitalized for HF, most take multiple FRIDs, and more than half of older adults with HF have a high FRID burden at hospital discharge. This work has important implications for the care of this population.

Our finding that FRIDs are nearly universal among older adults hospitalized for HF is important because FRIDs can potentially increase the risk for falls through their effects on the cardiovascular and central nervous systems [18, 19]. FRIDs may be especially problematic for older adults with HF, a subpopulation with an elevated risk for falls [2, 34, 35]. In a recent study of ambulatory patients with HF, 39% reported at least one fall in the prior year and 25% reported at least two falls in the prior year [36]. The risk of falls is further elevated following an HF hospitalization, as data from the Rehabilitation Therapy in Older Acute Heart Failure Patients (REHAB-HF) trial showed that the prevalence of falls prior to an HF hospitalization was 15%, and increased to 53% during the next 6 months [37]. Falls often lead to injuries and are associated with significant morbidity and mortality [38, 39]. Falls also have a significant psychological impact, leading to fear and anxiety surrounding future falls with related impairments in well-being and quality of life [39]. Given their highly prevalent use and potential impact on falls, FRIDs should become part of the usual lexicon for clinicians caring for older adults with HF.

FRIDs could be a potential target for deprescribing as a strategy to improve outcomes in older adults with HF. However, simply stopping or refraining from prescribing FRIDs may not be a simple decision. We found that the majority of FRIDs taken by adults with HF were CV in nature, and that many of these agents are recommended by clinical practice guidelines given their potential long-term benefit. For example, the most commonly initiated FRID on discharge was loop diuretics, which have a class I recommendation for treating HF [40]. Renin-angiotensin system inhibitors (beta-blockers, ACEi) are also beneficial for selected subtypes of HF. This creates a clinical conundrum. These agents are recommended given their potential to improve symptoms and/or long-term outcomes, but can increase the risk of falls via hypovolemia, hypotension, and/or bradycardia [41]. However, the risk of falls conferred by CV medications appears to be less than for non-CV drugs. Indeed, in a meta-analysis assessing medication use and falls in older adults, CV drugs (antihypertensives, diuretics) were less likely to cause falls compared to psychotropic drugs (sedatives/hypnotics, antidepressants, and benzodiazepines) [42]. As it may not be feasible to deprescribe CV medications given their clinical indication for HF, clinicians may instead consider regularly reviewing patient’s non-CV medications and evaluate their indication and side effect profile. On the whole, there is insufficient data to either support or refute the potential benefit of deprescribing FRIDs as a routine strategy to reduce fall risk in older adults. Therefore, decisions on how to address FRIDs use, such as potential dose optimization, in older adults with HF should be viewed as a preference-sensitive decision that requires shared decision-making and consideration of specific patient features (such as the presence of symptoms like hypotension or dizziness) [43]. Toward this end, several prescribing tools exist to aid clinicians in optimizing FRIDs use in the elderly, such as the STOPP/START (Screening Tool of Older Persons Prescriptions/Screening Tool to Alert to Right Treatment) tool, the Beers criteria, and the FORTA (Fit fOR The Aged) classification system [44–46]. Given their potential to reduce falls, they may be worth incorporating into routine clinical care of older adults with HF as part of a multifactorial falls prevention strategy [47, 48].

It may be important to consider FRID burden when making clinical decisions about medications. In this study, we used a tool for calculating FRID burden that is available on the AHRQ website. Although this tool requires further validation, it provides a convenient method for clinicians to quantify potential medication-related fall risk. It may be reasonable to use this tool as a starting point for a more comprehensive assessment to further assess overall fall risk in older adults with HF, especially among those with hyperpolypharmacy—a strong correlate for having a high FRIDs burden [49]. By identifying these patients, clinicians can then recommend select, targeted interventions to reduce falls risk. In addition, evaluating cognition, frailty, and gait may accordingly be an important strategy among those with hyperpolypharmacy given their association with falls [9, 50, 51]. At the very least, patients with a high FRID burden should be asked if they have a history of falls to identify risk, with subsequent incorporation into the risk and potential benefits of FRIDs.

Given the inherent risk of falls among older adults hospitalized for HF is in part attributable to the nearly universal use of FRIDs, it may be reasonable to routinely implement selected interventions for reducing the risk of falls. These interventions should include regular visits with primary care physicians to assess frailty status and gait speed as measures of functional ability. Clinicians can also provide patients practical tips to reduce falls risk, such as staying physically active, illuminating stairs and minimizing clutter in the household, wearing comfortable shoes, and using durable medical equipment such as canes or walkers. The World Falls Guidelines for falls prevention and management also emphasizes the importance of exercise programs including functional exercises (i.e.: sit-to-stand, stepping) and individualized strength training [52]. These exercises may be performed under the guidance and supervision of appropriately trained professionals, such as physiotherapists or kinesiologists, who can personalize them to the patients' functional status. Additional measures may involve employing home support services can help to create a safe environment, such as through the use of shower grab bars, handrails, and nonslip mats, and to implement quality home-fall hazard interventions. Occupational therapists may also help to assess environmental hazards and to determine the patients’ functional capacity in the context of their home environment. Regular vision assessments can also help to reduce fall risk [53]. Together, these measures are key to reduce falls and promote well-being, and thus should be part of a comprehensive care plan for many older adults with HF.

As outlined by a recent American Heart Association scientific statement, there is still much work to be done in the area of falls [1]. Few prospective studies have focused on identifying fall risk in adults with cardiovascular disease. Our study here serves as a foundation for future work examining the intersection of FRIDs and falls among older adults with HF, and for the development of individualized fall prevention interventions to meet the unique needs of this growing subpopulation of older adults living with HF.

This study has several strengths. First, REGARDS is a geographically diverse cohort of participants with HF treated in hospitals from all 48 contiguous United States. Therefore, this study has a high degree of generalizability specifically for older adults with HF. The availability of medical records was another strength of this study, as it allowed for detailed abstraction of participants’ medications and medical comorbidities at the time of hospitalization. Our study also has some limitations. First, we did not have sufficient data to calculate participant-level falls risk—it is likely that the risk of FRIDs is largely dependent on the participant-level falls risk. Accordingly, future work should focus on integrating FRID burden with participant-level falls risk. Second, we did not account for medication dosages or schedules, which likely impacts the medication-related risk for falls. Third, there is no universal list of FRIDs, and there is variation in the observed associations between various medications and falls. Different definitions could therefore lead to differing prevalence rates. We chose to examine a broad list of FRIDs based on prior comprehensive systematic reviews and meta-analyses [18, 19, 27–29]. Finally, while it is intuitive that higher scores would be associated with higher risk for falls, the AHRQ tool used in our study to quantify FRID burden has not yet been validated—this highlights yet another area ripe for future study.

## Conclusion

In conclusion, our study showed that FRIDs were nearly universal among older adults hospitalized for HF, and more than half had a high FRID burden. Moreover, most of these FRIDs were CV in nature. This underscores the need for clinicians to consider fall risk in decision-making especially as it relates to medications.

## Supplementary Information

**Additional file 1:** **Supplemental Table 1.** Baseline Characteristics According to FRIDs use at Admission. **Supplemental Table 2.** Baseline Characteristics According to FRIDs use at Discharge. **Supplemental Table 3.** Most Commonly Initiated and Discontinued FRIDs, Stratified by HF Subtype. **Supplemental Table 4.** Prevalence of FRIDs Use at Hospital Admission and Discharge Among Those With HFpEF. **Supplemental Table 5.** Prevalence of FRIDs Use at Hospital Admission and Discharge Among Those With HFrEF. **Supplemental Figure 1.** Frequency of FRID Counts for HFpEF. **Supplemental Figure 2.** Frequency of FRID Counts for HFrEF. **Supplemental Figure 3.** Frequency of AHRQ Fall Risk Score for HFpEF. **Supplemental Figure 4.** Frequency of AHRQ Fall Risk Score for HFrEF.

## Data Availability

Data was obtained from the REGARDS study, a private database. The datasets generated and/or analyzed during the current study are not publicly available, as the data includes characteristics that may compromise individual patient privacy; but are available from the corresponding author on reasonable request.
