# Supplementary material for: Prescribing patterns of fall risk-increasing drugs in older adults hospitalized for heart failure
Source: BMC Cardiovasc Disord. 2023 Jul 26;23:372. doi: 10.1186/s12872-023-03401-w (PMC10373421; doi:10.1186/s12872-023-03401-w)

Supplemental Table 1: Baseline Characteristics According to FRIDs use at Admission

| **Characteristics** | **Count of FRIDS** | | | |
| --- | --- | --- | --- | --- |
|  | 1 | 2 | 3 | ≥4 |
| N | 139 | 225 | 283 | 433 |
| Age, years | 79.1 (72.5, 84.4) | 79.1 (73.2, 84.3) | 78.5 (72.3, 83.8) | 76.1 (71.3, 81.8) |
| Black | 45 (32.4%) | 82 (36.4%) | 119 (42.0%) | 192 (44.3%) |
| Female | 58 (41.7%) | 99 (44.0%) | 141 (49.8%) | 209 (48.3%) |
| Education less than high school | 20 (14.4%) | 52 (23.1%) | 60 (21.2%) | 95 (21.9%) |
| Income <$20,000 | 35 (28.0%) | 56 (28.3%) | 67 (27.8%) | 113 (29.8%) |
| **Clinical Characteristics** |  |  |  |  |
| HFrEF | 45 (54.2%) | 81 (57.9%) | 91 (49.7%) | 158 (52.5%) |
| Hypertension | 97 (69.8%) | 162 (72.0%) | 233 (82.3%) | 381 (88.2%) |
| Atrial fibrillation/atrial flutter | 49 (35.3%) | 94 (41.8%) | 121 (42.8%) | 189 (43.6%) |
| Mood disorder | 16 (11.5%) | 30 (13.3%) | 39 (13.8%) | 88 (20.3%) |
| Osteoarthritis | 37 (26.6%) | 57 (25.3%) | 78 (27.6%) | 115 (26.6%) |
| Rheumatoid arthritis | 3 (2.2%) | 5 (2.2%) | 5 (1.8%) | 9 (2.1%) |
| Cancer | 33 (23.7%) | 42 (18.7%) | 55 (19.4%) | 80 (18.5%) |
| Anticoagulant use | 29 (20.9%) | 72 (32.0%) | 90 (31.8%) | 137 (31.6%) |
| Hyperpolypharmacy (at discharge) | 52 (37.4%) | 98 (43.6%) | 162 (57.2%) | 325 (75.1%) |
| Functional impairment | 47 (36.2%) | 80 (37.9%) | 111 (41.3%) | 210 (52.4%) |
| Cognitive impairment | 12 (11.9%) | 32 (19.8%) | 30 (14.2%) | 45 (14.1%) |
| History of falls (in the past year) | 27 (19.4%) | 44 (19.6%) | 55 (19.4%) | 98 (22.8%) |
| **Hospitalization Factors** |  |  |  |  |
| Length of stay (days) | 5.00 (3.0, 8.0) | 5.0 (3.0, 7.0) | 5.0 (3.0, 8.0) | 5.0 (3.0, 7.0) |
| ICU stay during hospitalization | 36 (25.9%) | 38 (17.0%) | 44 (15.5%) | 73 (16.9%) |
| Geriatric/palliative involvement | 3 (2.2%) | 1 (0.4%) | 3 (1.1%) | 5 (1.2%) |
| Disposition |  |  |  |  |
| Home | 107 (77.0%) | 182 (80.9%) | 221 (78.1%) | 346 (79.9%) |
| Institution | 28 (20.1%) | 34 (15.1%) | 46 (16.3%) | 68 (15.7%) |
| Alive, unknown disposition | 4 (2.9%) | 9 (4.0%) | 16 (5.7%) | 19 (4.4%) |
| **Hospital Characteristics** |  |  |  |  |
| Teaching status | 59 (48.4%) | 106 (53.0%) | 138 (53.1%) | 193 (50.9%) |
| Small hospital size (<200 beds) | 34 (26.8%) | 49 (24.0%) | 68 (25.9%) | 98 (25.3%) |

Values are n (%), unless otherwise indicated.

Abbreviations:

HF = heart failure, HFrEF = heart failure with reduced ejection fraction

ICU = intensive care unit

Supplemental Table 2: Baseline Characteristics According to FRIDs use at Discharge

| **Characteristics** | **Count of FRIDs** | | | |
| --- | --- | --- | --- | --- |
|  | 1 | 2 | 3 | ≥4 |
| N | 83 | 199 | 321 | 529 |
| Age, years | 76.8 (71.3, 83.5) | 79.4 (74.0, 83.9) | 78.3 (72.6, 83.4) | 76.5 (71.6, 82.3) |
| Black | 22 (26.5%) | 65 (32.7%) | 127 (39.6%) | 237 (44.8%) |
| Female | 33 (39.8%) | 79 (39.7%) | 159 (49.5%) | 253 (47.8%) |
| Education less than high school | 14 (16.9%) | 40 (20.1%) | 70 (21.8%) | 116 (21.9%) |
| Income <$20,000 | 17 (22.4%) | 48 (28.1%) | 78 (27.5%) | 138 (30.0%) |
| **Clinical Characteristics** |  |  |  |  |
| HFrEF | 23 (44.2%) | 69 (57.5%) | 110 (55.8%) | 191 (52.0%) |
| Hypertension | 53 (63.9%) | 134 (67.7%) | 251 (78.4%) | 455 (86.0%) |
| Atrial fibrillation/atrial flutter | 37 (44.6%) | 87 (43.7%) | 133 (41.4%) | 218 (41.2%) |
| Mood disorder | 6 (7.2%) | 21 (10.6%) | 39 (12.1%) | 106 (20.0%) |
| Osteoarthritis | 27 (32.5%) | 43 (21.6%) | 84 (26.2%) | 140 (26.5%) |
| Rheumatoid arthritis | 0 (0.0%) | 8 (4.0%) | 10 (3.1%) | 8 (1.5%) |
| Cancer | 18 (21.7%) | 37 (18.6%) | 53 (16.5%) | 108 (20.4%) |
| Anticoagulant use | 28 (33.7%) | 60 (30.2%) | 103 (32.1%) | 156 (29.5%) |
| Hyperpolypharmacy (at discharge) | 21 (25.3%) | 71 (35.7%) | 152 (47.4%) | 410 (77.5%) |
| Functional impairment | 23 (29.1%) | 70 (38.5%) | 124 (40.9%) | 238 (48.1%) |
| Cognitive impairment | 7 (11.5%) | 21 (15.4%) | 37 (15.7%) | 58 (14.6%) |
| History of falls (in the past year) | 15 (18.1%) | 37 (18.7%) | 60 (18.7%) | 119 (22.6%) |
| **Hospitalization Factors** |  |  |  |  |
| Length of stay (days) | 6.0 (4.0, 10.0) | 5.0 (3.0, 8.0) | 5.0 (3.0, 8.0) | 4.0 (3.0, 7.0) |
| ICU stay during hospitalization | 20 (24.1%) | 39 (19.6%) | 55 (17.2%) | 89 (16.9%) |
| Geriatric/palliative involvement | 1 (1.2%) | 4 (2.0%) | 2 (0.6%) | 5 (0.9%) |
| Disposition |  |  |  |  |
| Home | 64 (77.1%) | 167 (83.9%) | 249 (77.6%) | 419 (79.2%) |
| Institution | 18 (21.7%) | 25 (12.6%) | 59 (18.4%) | 79 (14.9%) |
| Alive, unknown disposition | 1 (1.2%) | 7 (3.5%) | 13 (4.1%) | 31 (5.9%) |
| **Hospital Characteristics** |  |  |  |  |
| Teaching status | 42 (56.0%) | 97 (55.1%) | 144 (49.3%) | 237 (50.9%) |
| Small hospital size (<200 beds) | 17 (22.4%) | 43 (23.4%) | 89 (30.3%) | 110 (23.2%) |

Values are n (%), unless otherwise indicated.

Abbreviations:

HF = heart failure, HFrEF = heart failure with reduced ejection fraction

ICU = intensive care unit

Supplemental Table 3: Most Commonly Initiated and Discontinued FRIDs, Stratified by HF Subtype

|  | **FRID** | **n (%)** |
| --- | --- | --- |
| **Initiated** |  |  |
| *HFpEF* | 1. Loop diuretics | 91 (26) |
|  | 2. Beta blockers | 40 (11) |
| *HFrEF* | 1. Loop diuretics | 103 (26) |
|  | 2. Beta blockers | 87 (22) |
| **Discontinued** |  |  |
| *HFpEF* | 1. Thiazides | 38 (11) |
|  | 2. CCBs | 31 (9) |
| *HFrEF* | 1. CCBs | 50 (13) |
|  | 2. Thiazides | 37 (9) |

Abbreviations:

HFpEF = heart failure with preserved ejection fraction

HFrEF = heart failure with reduced ejection fraction

CCB = calcium channel blocker

Supplemental Table 4: Prevalence of FRIDs Use at Hospital Admission and Discharge Among Those With HFpEF

|  | **Admission** | **Discharge** | **Change** |
| --- | --- | --- | --- |
| **All FRIDs** | 1080 (94) | 1132 (99) | 5% |
| **CV FRIDs** | 1060 (92) | 1122 (98) | 6% |
| **Antihypertensives** | 1005 (88) | 1072 (93) | 5% |
| Beta blockers | 700 (61) | 863 (75) | 14% |
| ACE inhibitors | 411 (36) | 480 (42) | 6% |
| CCBs | 369 (32) | 325 (28) | -4% |
| Nitrates | 179 (16) | 250 (22) | 6% |
| Alpha blockers | 230 (20) | 215 (19) | 1% |
| ARBs | 228 (20) | 203 (18) | -2% |
| Vasodilators | 99 (9) | 152 (13) | 4% |
| Aldosterone antagonists | 81 (7) | 148 (13) | 6% |
| Thiazides | 183 (16) | 90 (8) | -8% |
| Potassium-sparing diuretics | 23 (2) | 8 (1) | -1% |
| Alpha agonists | 7 (1) | 9 (1) | - |
| Loop diuretics | 612 (53) | 829 (72) | 19% |
| Digoxin | 141 (12) | 185 (16) | 4% |
| **Non-CV FRIDs** | 369 (32) | 368 (32) | - |
| **Antidepressants** | 234 (20) | 243 (21) | 1% |
| SSRI | 157 (14) | 165 (14) | - |
| TCA | 46 (4) | 46 (4) | - |
| SNRI | 28 (2) | 30 (3) | 1% |
| Trazodone | 10 (1) | 12 (1) | - |
| NDRI | 8 (1) | 8 (1) | - |
| Benzodiazepines | 93 (8) | 87 (8) | - |
| Opioids | 97 (8) | 71 (6) | -2% |
| Antiepileptics | 30 (3) | 34 (3) | - |
| **Antipsychotics** | 19 (2) | 29 (2) | - |
| Atypical | 16 (1) | 24 (2) | 1% |
| Typical | 3 (0) | 5 (0) | - |

Abbreviations:

HFpEF = heart failure with preserved ejection fraction

FRIDs = fall risk-increasing drugs

CV = cardiovascular

ACEi = angiotensin-converting enzyme inhibitors

CCB = calcium channel blocker

ARB = angiotensin receptor blocker

Non-CV = non-cardiovascular

SSRI = selective serotonin reuptake inhibitor

TCA = tricyclic antidepressant

SNRI = serotonin and norepinephrine reuptake inhibitor

NDRI = norepinephrine and dopamine reuptake inhibitor

Supplemental Table 5: Prevalence of FRIDs Use at Hospital Admission and Discharge Among Those With HFrEF

|  | **Admission** | **Discharge** | **Change** |
| --- | --- | --- | --- |
| **All FRIDs** | 1080 (94) | 1132 (99) | 5% |
| **CV FRIDs** | 1060 (92) | 1122 (98) | 6% |
| **Antihypertensives** | 1005 (88) | 1072 (93) | 5% |
| Beta blockers | 700 (61) | 863 (75) | 14% |
| ACE inhibitors | 411 (36) | 480 (42) | 6% |
| CCBs | 369 (32) | 325 (28) | -4% |
| Nitrates | 179 (16) | 250 (22) | 6% |
| Alpha blockers | 230 (20) | 215 (19) | 1% |
| ARBs | 228 (20) | 203 (18) | -2% |
| Vasodilators | 99 (9) | 152 (13) | 4% |
| Aldosterone antagonists | 81 (7) | 148 (13) | 6% |
| Thiazides | 183 (16) | 90 (8) | -8% |
| Potassium-sparing diuretics | 23 (2) | 8 (1) | -1% |
| Alpha agonists | 7 (1) | 9 (1) | - |
| Loop diuretics | 612 (53) | 829 (72) | 19% |
| Digoxin | 141 (12) | 185 (16) | 4% |
| **Non-CV FRIDs** | 369 (32) | 368 (32) | - |
| **Antidepressants** | 234 (20) | 243 (21) | 1% |
| SSRI | 157 (14) | 165 (14) | - |
| TCA | 46 (4) | 46 (4) | - |
| SNRI | 28 (2) | 30 (3) | 1% |
| Trazodone | 10 (1) | 12 (1) | - |
| NDRI | 8 (1) | 8 (1) | - |
| Benzodiazepines | 93 (8) | 87 (8) | - |
| Opioids | 97 (8) | 71 (6) | -2% |
| Antiepileptics | 30 (3) | 34 (3) | - |
| **Antipsychotics** | 19 (2) | 29 (2) | - |
| Atypical | 16 (1) | 24 (2) | 1% |
| Typical | 3 (0) | 5 (0) | - |

Abbreviations:

HFrEF = heart failure with reduced ejection fraction

FRIDs = fall risk-increasing drugs

CV = cardiovascular

ACEi = angiotensin-converting enzyme inhibitors

CCB = calcium channel blocker

ARB = angiotensin receptor blocker

Non-CV = non-cardiovascular

SSRI = selective serotonin reuptake inhibitor

TCA = tricyclic antidepressant

SNRI = serotonin and norepinephrine reuptake inhibitor

NDRI = norepinephrine and dopamine reuptake inhibitor

Supplemental Figure 1: Frequency of FRID Counts for HFpEF


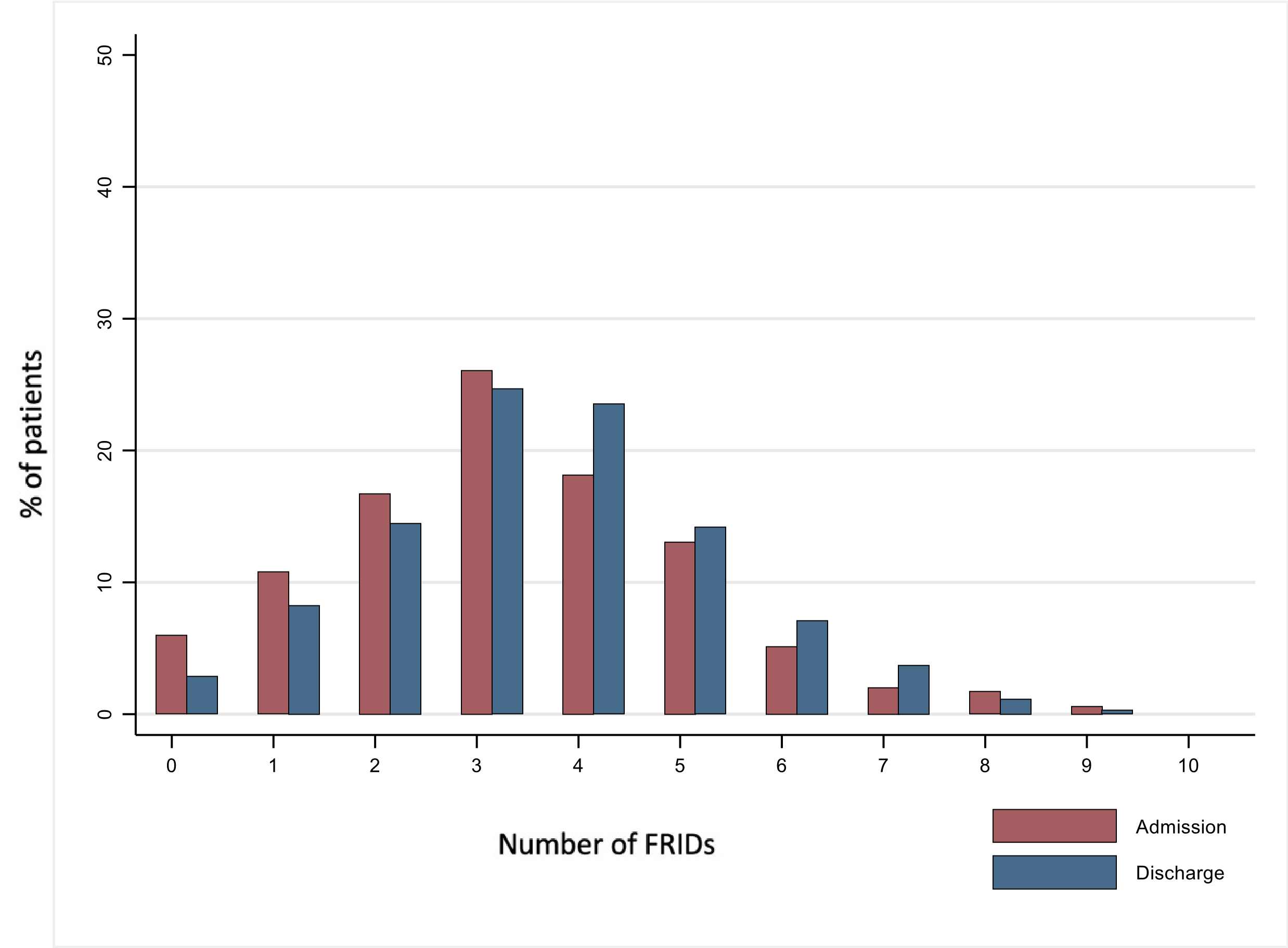


Supplemental Figure 2: Frequency of FRID Counts for HFrEF


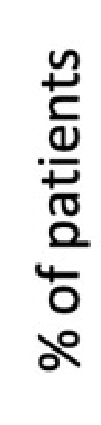

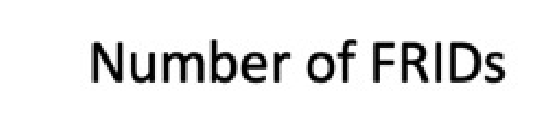


Supplemental Figure 3: Frequency of AHRQ Fall Risk Score for HFpEF


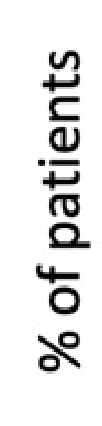

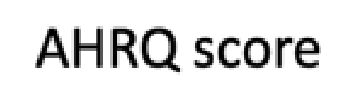


Supplemental Figure 4: Frequency of AHRQ Fall Risk Score for HFrEF


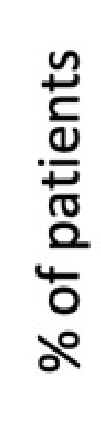

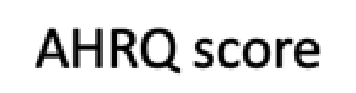

Supplement: Supplementary file 1 — Additional file 1: Supplemental Table 1. Baseline Characteristics According to FRIDs use at Admission. Supplemental Table 2. Baseline Characteristics According to FRIDs use at Discharge. Supplemental Table 3. Most Commonly Initiated and Discontinued FRIDs, Stratified by HF Subtype. Supplemental Table 4. Prevalence of FRIDs Use at Hospital Admission and Discharge Among Those With HFpEF. Supplemental Table 5. Prevalence of FRIDs Use at Hospital Admission and Discharge Among Those With HFrEF. Supplemental Figure 1. Frequency of FRID Counts for HFpEF. Supplemental Figure 2. Frequency of FRID Counts for HFrEF. Supplemental Figure 3. Frequency of AHRQ Fall Risk Score for HFpEF. Supplemental Figure 4. Frequency of AHRQ Fall Risk Score for HFrEF. [file 12872_2023_3401_MOESM1_ESM.docx]
